# Antithyroid Drug Therapy for Graves' Disease and Implications for Recurrence

**DOI:** 10.1155/2017/3813540

**Published:** 2017-04-25

**Authors:** Jia Liu, Jing Fu, Yuan Xu, Guang Wang

**Affiliations:** Department of Endocrinology, Beijing Chao-Yang Hospital, Capital Medical University, Beijing 100020, China

## Abstract

Graves' disease (GD) is the most common cause of hyperthyroidism worldwide. Current therapeutic options for GD include antithyroid drugs (ATD), radioactive iodine, and thyroidectomy. ATD treatment is generally well accepted by patients and clinicians due to some advantages including normalizing thyroid function in a short time, hardly causing hypothyroidism, and ameliorating immune disorder while avoiding radiation exposure and invasive procedures. However, the relatively high recurrence rate is a major concern for ATD treatment, which is associated with multiple influencing factors like clinical characteristics, treatment strategies, and genetic and environmental factors. Of these influencing factors, some are modifiable but some are nonmodifiable. The recurrence risk can be reduced by adjusting the modifiable factors as much as possible. The titration regimen for 12–18 months is the optimal strategy of ATD. Levothyroxine administration after successful ATD treatment was not recommended. The addition of immunosuppressive drugs might be helpful to decrease the recurrence rate of GD patients after ATD withdrawal, whereas further studies are needed to address the safety and efficacy. This paper reviewed the current knowledge of ATD treatment and mainly focused on influencing factors for recurrence in GD patients with ATD treatment.

## 1. Introduction

Graves' disease (GD) is an organ-specific autoimmune disease characterized as overproduction of thyroid hormones in thyroid follicular cells resulting from the stimulation of circulating thyroid-stimulating hormone (TSH) receptor antibodies (TRAb) [1]. It is the most common cause of hyperthyroidism worldwide [1]. Current therapeutic options for GD include antithyroid drugs (ATD), radioactive iodine, and thyroidectomy [1, 2]. ATD treatment has many advantages, including normalizing thyroid function in a short time, hardly causing hypothyroidism, and ameliorating immune disorder while avoiding radiation exposure and invasive procedures, so it is generally well accepted by patients and clinicians [3, 4]. However, the high recurrence rate is a main limitation of ATD treatment, which varies greatly among patients with different treatment strategies, clinical characteristics, and environmental and genetic factors [2, 5–8]. In this paper, we review the current knowledge of ATD treatment and mainly focus on influencing factors for recurrence in GD patients with ATD treatment.

## 2. Treatment Strategies of ATD and the Recurrence Risk

### 2.1. Drug Selection

The commonly used ATD include methimazole and propylthiouracil [1]. Carbimazole is another ATD that is available in few regions [9–11]. Carbimazole exerts its pharmacological effect by converting to methimazole, so it has similar efficacy and features as methimazole [9–11]. Previous studies showed that methimazole had a better efficacy and restored the euthyroid state much faster than propylthiouracil, but the recurrence rate after withdrawal was comparable between the two drugs in GD patients [12–14]. The side effects of ATD is common (13%) but generally mild, including rash, pruritus, metallic taste, arthralgia, and liver damage [2]. A meta-analysis of 31 observational studies showed that rash was more common with methimazole treatment, whereas the predominant side effect of propylthiouracil was hepatic involvement [2]. ATD treatment also had some major side effects, including agranulocytosis and severe hepatotoxicity, which are life threatening but rare (<0.5%) [2]. Methimazole has longer half-life and duration of action and fewer major side effects as compared to propylthiouracil [2, 4]. Thus, methimazole is recommended as the preferred drug for GD patients by ATA guidelines except for pregnant women during the first trimester [2, 4, 15].

### 2.2. Treatment Regimen

There are two regimens of ATD: titration-block and block-replace regimens [16]. The titration-block regimen means that the ATD dose is titrated from the initial dose to the lowest dose for maintaining a euthyroid state, and the block-replace regimen is initiated with a standard dose of ATD and the addition of levothyroxine [16]. The initial dose of ATD depends on the severity of hyperthyroidism [12]. Patients with mild hyperthyroidism begin with 10–15 mg daily of methimazole, while 20–40 mg daily is given to patients with severe hyperthyroidism [12]. A recent meta-analysis from Cochrane showed that the two regimens had similar recurrence rates, but the block-replace regimens caused relatively more side effects [16].

### 2.3. Treatment Duration

Just as important, the treatment duration of ATD also impacts the recurrence risk of GD patients [7, 11, 17, 18]. About 20 years ago, most GD patients were treated by ATD for 6 months [11, 17]. Recently, increasing evidence has demonstrated that ATD treatment for 12–18 months leads to a better prognosis than the 6-month treatment [7, 18]. The results from a recent meta-analysis showed that the 12-month titration regimen has a lower recurrence rate than the 6-month regimen, but extending treatment beyond 18 months failed to provide more benefits [16]. In addition, considering the high recurrence rate after drug withdrawal, some studies even advocated a continuous treatment with low-dose ATD for GD patients [19, 20]. They proposed that long-term maintenance of low-dose ATD had a persistent effect on preventing recurrence [19, 20]. However, because of the intermittent blood check and higher medical costs during long-term ATD maintenance, the titration regimen for 12–18 months is still considered as the optimal strategy of ATD.

### 2.4. Levothyroxine Administration after Successful ATD Treatment

Because increased TSH levels have been incriminated for promoting the production of TRAb, some studies have been conducted to evaluate whether levothyroxine administration after successful ATD treatment could decrease the recurrence risk of hyperthyroidism in GD patients [21–23]. Nevertheless, these studies demonstrated that levothyroxine does not prevent recurrence of hyperthyroidism in GD patients after successful ATD treatment [21, 22]. Mastorakos et al. even observed that levothyroxine administration after successful ATD treatment was associated with increased recurrence risk of GD patients [23]. Thus, levothyroxine administration after successful ATD treatment was not recommended.

### 2.5. Additional Use of Immunosuppressive Drugs for Standard ATD Treatment

Considering the autoimmune nature of GD, several studies have attempted to evaluate the effect of additional use of immunosuppressive drugs on ATD treatment in GD patients [24]. The immunosuppressive drugs for GD patients mainly included corticosteroid and noncorticosteroid drugs [25–29]. A recent meta-analysis demonstrated a strong reduction of the recurrence risk when immunosuppressive drugs were added to standard ATD treatment in GD patients, and the reduction of the recurrence risk was similar in studies using corticosteroid and noncorticosteroid immunosuppressive drugs [30]. In this meta-analysis, the overall recurrence rate in GD patients receiving the addition of immunosuppressive drugs was 23.5%, which was significantly lower than 59.1% in GD patients only treated with ATD [30]. Therefore, the addition of immunosuppressive drugs might be helpful to decrease the recurrence rate of GD patients after ATD withdrawal, whereas there are still some problems to mention. First, studies addressing immunosuppressive treatment for GD are always small, single-center, and with low to moderate quality and high risk of bias [25–29]. Second, the side effects of immunosuppressive drugs should have great importance [30]. The administration mode of immunosuppressive drug included oral and local administrations [25–29]. However, the side effects should not be disregarded, no matter the mode [30]. The side effects of corticosteroids include bone abnormalities, metabolic disturbances, and muscle wasting, and rituximab was associated with leucopenia, rash, minor infections, chills, and fever [28, 30]. Therefore, further large-scale randomized controlled trials are needed to address the safety, efficacy, optimal timing, and duration of immunosuppressive drugs in GD patients.

## 3. Influencing Factors for the Recurrence in GD Patients

### 3.1. General Situation

The incidence of GD gradually increases with age and then remains stable after the age of 30 [31]. It is generally accepted that younger GD patients have more severe immune disorders [32]. Previous studies showed that younger GD patients have a relatively poor response to ATD and often have a poor prognosis and higher recurrence risk [5, 33, 34]. A study by Allahabadia et al. found that GD patients younger than 40 years had a higher recurrence rate than older patients [34].

Females have a higher incidence of GD than males [31, 35]. The reason for a different incidence in gender is unclear and might be associated with varying sex hormones [36]. Estrogens influenced B-cell function and further regulated the immune system [36]. In GD patients, the increased estradiol level is related to the positivity of TRAb [37]. Although the incidence of GD is higher in women, male GD patients have a higher risk of recurrence after ATD withdrawal [6, 34, 38, 39]. There are some possible explanations for the higher recurrence risk of male GD patients. First, male GD patients had larger goiter size, which was associated with severe immune and biochemical disorders [39]. Second, a family history of thyroid or nonthyroid autoimmune diseases occurred more frequently in male GD patients [39]. So, the higher risk of recurrence in male GD patients might be associated with bigger goiter size and genetic background. However, this is still a conflicting issue since other studies have inconsistent results [8, 40].

Several studies showed that smokers have a higher recurrence risk than nonsmokers in GD patients after ATD withdrawal [38, 41–43]. Meanwhile, quitting smoking has also been demonstrated to protect against recurrence in GD patients [40]. A previous study showed that smokers had significant higher TRAb levels than nonsmokers at 4 weeks after ATD withdrawal [44]. Therefore, smoking might promote immune disorder and elevate TRAb levels, contributing to the increased recurrence risk. Because there were some inconsistent results, the association between smoking and the recurrence rate still remains uncertain [8].

### 3.2. Biochemical Parameter

The severity of GD is associated with the recurrence risk in GD patients treated with ATD [8, 45–47]. The biochemical parameters partially represent the severity of GD [8, 45–47]. The key feature in untreated GD is the significant increase in the serum triiodothyronine (T3) level, which is caused by the elevated activity of intrathyroidal type 1 deiodinase [1]. Previous studies showed that the serum T3 levels and free T3 (FT3)/free thyroxin (FT4) ratios at the onset of GD were independent factors for predicting outcomes of ATD treatment in GD patients [8, 45–47]. Patients with mild hyperthyroidism can achieve remission after just treatment with beta blockers [48]. However, patients with higher serum T3 levels and FT3/FT4 ratios have a relatively higher recurrence risk, so they often need a higher initial dose and longer treatment duration [45–47]. Furthermore, a high T3/T4 ratio during ATD withdrawal also predicts a higher recurrence risk in GD patients [4]. The therapy duration should be prolonged in patients with a high T3/T4 ratio even after 12–18 months of ATD treatment.

In addition, the serum TSH level also should get more attention. As is known, the thyroid hormone can inhibit TSH via a negative feedback mechanism [49]. In some GD patients, the TSH level still sustained suppression and failed to return to a normal range with the normalization of thyroid hormone after ATD treatment [38, 49]. Previous studies have demonstrated that TSH suppression after drug withdrawal was a predictor for the recurrence of GD [8, 38, 50]. These studies suggested that GD patients with delayed TSH restoration should receive prolonged ATD treatment until their TSH levels reach the normal range.

### 3.3. Immune Parameters

As mentioned before, GD results from the overactivated TSH receptor in the thyroid follicular cells by TRAb [1]. TRAb is positive in about 95% of patients with newly diagnosed GD, and the higher TRAb levels hint a severe immune disorder [1, 5, 14, 51, 52]. In recent years, with increased accuracy of assay, TRAb has been supported as a useful predictive factor for the outcome of ATD treatment by many studies [5, 14, 51, 52]. Patients with higher TRAb levels at the time of GD diagnosis have significantly increased recurrence risk, while TRAb-negative patients often have a better prognosis and are prone to long-term remission [5, 14, 52]. Furthermore, a shift from positive to negative in TRAb may imply the alleviated immune disorder following ATD treatment in GD patients [41, 43, 50]. The TRAb level at ATD withdrawal was also associated with the prognosis of GD patients [41, 43, 50]. The recurrence risk was higher in TRAb-positive GD patients at the time of drug withdrawal [41, 43, 50]. In addition, TRAb can be distinguished from the stimulating (TSAb) and blocking (TBAb) properties by using new assay techniques [53]. Antibodies of GD patients are predominantly TSAb [1]. Recently, TSAb has been demonstrated to have a superior predictive value for recurrence risk than TRAb in GD patients treated with ATD [53–55].

GD and Hashimoto's thyroiditis are the two main autoimmune thyroid diseases [56]. Prior epidemiologic studies have indicated that GD is possibly concomitant with Hashimoto's thyroiditis [57]. GD patients with Hashimoto's thyroiditis tend to be in remission after ATD treatment due to the progressive damage induced by Hashimoto's thyroiditis [57]. Since the positivity of peroxidase autoantibodies (TPOAb) and/or thyroglobulin antibody (TgAb) is the main feature of Hashimoto's thyroiditis, some studies have evaluated the association between the positivity of TgAb/TPOAb and the recurrence risk in GD patients [58, 59]. The reason for the inconsistent results might be that both TPOAb and TgAb also could be observed in some GD patients without Hashimoto's thyroiditis [59].

### 3.4. Goiter Size

Large goiter size is a main clinical manifestation of GD patients [5, 8]. Previous studies have demonstrated that goiter size was a major predictor of a higher recurrence risk in GD patients after ATD withdrawal [5, 8]. A 5-year follow-up trail showed that patients with normal or mild goiter size had higher remission rates than patients with large goiters [49]. Moreover, GD patients with significantly decreased goiter sizes after ATD treatment also tend to have higher remission rates [41, 54]. These studies suggested that enlarged goiter size at the time of GD diagnosis and drug withdrawal is associated with a higher recurrence risk.

### 3.5. Graves' Orbitopathy

Graves' orbitopathy is present in around 30% of patients at the time of diagnosis of GD [40, 60]. The presence of Graves' orbitopathy often suggested a worse disorder of the immune system [60]. Previous studies have shown that patients with Graves' orbitopathy have a higher recurrence risk of GD after ATD withdrawal [38]. A study by Eckstein et al. even found that the remission rate of GD patients with severe Graves' orbitopathy was just 7% [61]. Even with the higher recurrence rate, ATD treatment is still a preferred therapeutic option for GD patients with Graves' orbitopathy due to a better outcome for Graves' orbitopathy, which might be associated with the stable euthyroid status and decreased levels of TRAb and inflammatory markers [62–65]. Recent studies have shown that a prolonged low dose of ATD treatment contributed to a better outcome in GD patients with Graves' orbitopathy [63, 66].

### 3.6. Genetic Factors

Genetic factors participate in the pathogenesis of GD [67]. Recent studies found that both cytotoxic T-lymphocyte-associated factor 4 (*CTLA4*) rs231775 and rs231779 polymorphisms were associated with recurrence in GD patients after ATD withdrawal in Asians, while no association was observed in Caucasians [5, 41, 68]. In Caucasian patients with GD, the recurrence risk after ATD withdrawal was reported to be related to the polymorphisms of HLA *DQA2*, HLA *DRB1*^∗^*03*, and HLA *DQB1*^∗^*02* [5]. The HLA region contains some immune response genes and these HLA polymorphisms might influence the outcome of GD patients by regulating the immune system. In addition, a few studies also explored the association between the recurrence risk and polymorphisms, including T393C SNP of Galphas gene (*GNAS1*), CD40 (rs745307, rs11569309, and rs3765457), and E33SNP of thyroglobulin (*Tg*) (*Tg* E33SNP) in GD patients after ATD withdrawal [41, 42, 69].

### 3.7. Environmental Factors

The onset of GD is induced by some environmental factors in the individuals with the predisposed gene [70]. Stress is one of the environmental factors, and the association between stress and the recurrence of GD patients after ATD treatment was supported by most studies [71–73]. A Japanese study showed that the 2004 earthquake was associated with the recurrence of GD in patients [74]. In a prospective study that evaluated the recurrence risk of GD in patients, a total stress score of big life events was significantly higher in the recurrence group than in the remission group [75]. Another study showed that the recurrence group experienced more stressful events than did remission patients, and the overall number of stressful events was correlated with the number of recurrences [71]. It is worth mentioning that psychosocial stress is an important part of a stressful event. Previous studies showed that GD patients with psychiatric disorders such as depression and hypochondriasis had a higher recurrence risk than GD patients without such disorders [75–77]. Thus, reducing stress as much as possible is an important way to improve the prognosis of GD patients with ATD treatment.

Iodine intake is another environmental factor [78]. Iodine is a major substrate for the synthesis of thyroid hormone [78]. In thyrocytes, increased iodine content promoted ATD degradation and reduced ATD uptake [79]. Some previous studies showed that iodine supplementation elevated the recurrence rate of GD [80]. The administration of pharmacological doses of iodine caused the onset of hyperthyroidism in euthyroid GD patients after ATD withdrawal [81]. However, epidemiologic studies showed that the recurrence rate in iodine-sufficient regions is not higher than that in iodine-deficient regions in GD patients after ATD withdrawal [40, 82, 83]. In a recent Korean study, excessive iodine did not influence the outcomes of GD [84]. The study suggested that dietary iodine restriction might not be necessary for GD patients after ATD withdrawal [84]. In addition, a previous study showed that a dietary change from a low-iodine to a high-iodine intake increased the recurrence rate in GD patients after ATD treatment [85]. Thus, these results might suggest that only a sudden increase in iodine intake induces the recurrence of GD. Further large-scale intervention studies are needed to evaluate the effect of iodine uptake on the recurrence risk in GD patients after ATD withdrawal.

In addition, baseline vitamin D and selenium levels might impact the outcome of GD patients after ATD treatment. Vitamin D has been demonstrated as an immune modulator [86]. An animal study showed that vitamin D regulated TSH receptor immunization in BALB/c mice [87]. And vitamin D analog also exhibited the inhibitory effect on inflammatory response in human thyroid cells and T cells [88]. ATD treatment caused a greater decline in TRAb in GD patients with normal vitamin D levels compared to that in GD patients with decreased vitamin D levels [89]. Moreover, a decreased baseline vitamin D level was associated with greater thyroid goiter in female patients with newly onset GD [90]. Selenium is another component element of thyroid function [91, 92]. As a basic component of glutathione peroxidase and iodothyronine selenodeiodinase, selenium deficiency might impact the conversion of T4 to T3 and the production of free radicals [91, 93]. The association between inadequate selenium supply and GD has been found by many studies [91, 92]. Furthermore, decreased serum selenium levels were also related to severe immune disorders and the incidence of Graves' orbitopathy [94, 95]. High serum selenium levels were also shown to be associated with higher remission rates in GD patients [96]. Recently, more studies have found that selenium supplementation can enhance biochemical restoration of hyperthyroidism and improve the remission rate of GD patients [93, 97]. It is worth noting that there have been relatively fewer studies to evaluate the association between the recurrence risk and the serum levels of vitamin D and selenium, and they were also small-scale, single-center studies. Further large-scale prospective studies are needed to observe this association and whether the administration of vitamin D and selenium can bring benefit.

## 4. Treatment Options for Recurrence GD Patients after ATD Withdrawal

The high recurrence rate is a major drawback of ATD therapy, and patients with recurrent GD often have a much higher recurrence risk than average [54, 82]. Most clinicians will recommend radioactive iodine or thyroidectomy for recurrent GD patients [98]. However, some recent studies have found that compared with radioactive iodine or thyroidectomy, the prolonged low-dose ATD treatment retained a stable euthyroid state while minimizing the risk of side effects [99, 100]. A recent prospective clinical study showed that ATD treatment also reached a higher remission rate in recurrent GD patients, and a much lower discontinuation drug dose of methimazole (2.5 mg qod) increased the rate of permanent remission [99]. In a recent study, during a long-term follow-up (up to 7 years), prolonged low-dose methimazole treatment was safe and effective and with fewer complications and less expense in recurrent GD patients [100]. It also contributes to a better outcome of Graves' orbitopathy and a lower frequency of thyroid dysfunction than radioactive iodine [100]. Thus, prolonged low-dose methimazole treatment might be a good alternative for recurrent GD patients who resist radioactive iodine or thyroidectomy.

## 5. Conclusions

Recurrence in GD patients with ATD treatment is associated with multiple influential factors such as clinical characteristics, treatment strategies, and genetic and environmental factors. Of these influencing factors, some are modifiable but some are nonmodifiable. The recurrence risk can be reduced by adjusting the modifiable factors as much as possible. If the recurrence evaluation based on the nonmodifiable factors strongly suggests a high risk of recurrence, a definitive treatment such as radioactive iodine or thyroidectomy is considered as an appropriate therapeutic approach. However, prolonged low-dose methimazole treatment might be a good alternative for GD patients with high recurrence risk due to its safety and efficacy. The addition of immunosuppressive drugs might be helpful to decrease the recurrence rate of GD patients after ATD withdrawal, whereas further studies are needed to address the safety and efficacy. Further large-scale prospective studies are also needed to observe whether the administration of vitamin D and selenium can bring benefit.
